# Diffusion-Weighted Imaging for the Discrimination of Benign and Malignant Breast Masses; Utility of ADC and Relative ADC

**DOI:** 10.5334/jbsr.1258

**Published:** 2018-02-07

**Authors:** Ebru Yılmaz, Ozcan Sarı, Ayhan Yılmaz, Nese Ucar, Ahmet Aslan, Ibrahim Inan, Ulku Tuba Parlakkılıc

**Affiliations:** 1 GOP Taksim Training and Educational Hospital, TR; 2 Bezmialem Vakif University Hospital, TR; 3 Medeniyet University Hospital, TR

**Keywords:** Breast MRI, Breast cancer, Diffusion-weighted imaging, Apparent diffusion coefficient (ADC), Relative ADC

## Abstract

**Purpose:**

To determine the contribution of apparent diffusion coefficient (ADC), and relative ADC (rADC) values to differentiate between benign and malignant breast masses.

**Materials and Methods:**

Magnetic resonance imaging (MRI) of the breast with diffusion-weighted imaging (DWI) of patients with benign or malignant breast masses diagnosed either by histopathological findings or by follow-up imaging were evaluated retrospectively. Histopathological analyses were performed for 71 lesions (80.7%) while the remaining were followed up every six months for one year. DWI was performed using b-values of 0 and 1000 sec/mm^2^, and ADC and rADC were calculated and compared. A receiver operating characteristic (ROC) curve and Youden index were used to evaluate the parameter’s optimal threshold and diagnostic value. Statistical significance was set as p < 0.05.

**Results:**

Eighty-eight lesions from a total of 81 patients, aged between 16 and 73 (mean age 42 ± 11.3) years were obtained and evaluated. Pathological results of 34 (38.6%) out of 71 lesions were malignant and 37 lesions (42%) were benign. Seventeen (19.3%) lesions remained stable at one-year follow-up and were accepted as benign breast masses. Mean ADC values of benign and malignant lesions were 1.584 × 10^–3^mm^2^/sec and 0.884 × 10^–3^mm^2^/sec (p < 0.05), respectively. Sensitivity and specificity of ADC were 88% and 87%, respectively at a cut-off value of 1.04 × 10^–3^mm^2^/sec. Mean rADC was 0.931 for benign lesions and 0.557 for malignant lesions (p < 0.05). Sensitivity and specificity were 82% and 83% at a cut-off value of 0.639. No prominent superiority of rADC over ADC is identified in the differentiation of breast masses.

**Conclusion:**

ADC and rADC values derived from DWI can be equally useful in clinical setting to differentiate benign from malignant breast masses.

## Introduction

Mammography remains the sole fundamental imaging method in diagnosis and screening of breast cancer [1]. Recently, the use of magnetic resonance imaging (MRI) as an addition to conventional methods in the diagnosis of primary or recurrent breast cancer has been gradually increasing [2]. Although breast MRI with contrast has a high diagnostic sensitivity for diagnosing the invasive breast cancer, the specificity of MRI is variable [3].

Advances in MRI technology (dedicated breast coils and quick imaging sequences) led to the inclusion of diffusion-weighted imaging (DWI) in breast studies by Englander et al. in 1997 [4]. Several recent studies indicated the utility of DWI that was subsequently included in routine breast MRI protocols in many centers. Unfortunately, the apparent diffusion coefficient (ADC) values obtained from DWI are affected by menstrual cycle and hormone replacement therapy [5,6]. Relative ADC (rADC) value is defined to optimize ADC value, which is calculated by dividing ADC value of the breast lesion by adjacent breast parenchyma. The rADC value is supposed to be unaffected by the menstrual cycle [7,8,9]. In this study, we introduced the rADC and ADC values to differentiate benign and malignant breast masses and compared their diagnostic performance.

## Material and Methods

The retrospective design of this study was approved by the local ethics committee and informed written consent was waived.

### Patient group

All breast MRI studies between the dates of November 2014 and May 2015 were screened retrospectively, and 421 patients were evaluated. Out of these patients, 128 patients had at least one breast mass. We excluded ten patients because of MRI assessment after biopsy, 19 patients in whom the histopathologic assessment was not available and/or performed in outer center, and 18 patients in whom the breast mass was not well-demarcated on DWI or assessable due to motion artifacts. Finally, a total of 81 patients were enrolled in the study.

### Magnetic resonance imaging

All examinations were performed with a 1.5 Tesla (T) MRI equipment (GE Signa HDx, GE Medical Systems, USA) using 8-channel phased array breast surface coil. Care was taken to perform the breast MRI of pre-menopausal women between the 5th and 15th days of the menstrual cycle. Conventional contrast MRI images were obtained with the following technique; axial fat-suppressed T2-weighted turbo spin-echo (repetition time/echo time) [TR/TE], 4500 msec/97 msec, 330 mm field of view [FOV], number of excitations (NEX): 1, matrix, 384 × 512; slice thickness of 3 mm with a 1 mm intersection gap and fat-suppressed T1-weighted (TR/TE, 720 msec/20 msec; 330 mm FOV, NEX: 2, matrix, 320 × 320; slice thickness of 3 mm) and fat-suppressed 3D T1-weighted images (4.3 ms/1.4 ms; flip-angle, 12°; a FOV of 320 mm; matrix, 307 × 512; signal average 1; slice thickness, 1.5 mm fast low angle shot (FLASH)) with and without contrast. Sagittal fat-supressed 3D T1-weighted images were obtained 6 minutes after contrast material injection in addition to axial dynamic contrast enhanced images. The contrast agent (Dotarem, Laboratoire Guerbet, Roissy, France) was administered as 0.2 mmol/kg via automatic syringe, followed by 15–20 cc of saline for homogeneous distribution of the contrast substance. DWI echo-planar images (TR/TE 8500/70, FOV of 330 mm, matrix 192 × 192, NEX:1, sectional thickness 4.5 mm with a 1 mm intersection gap) were obtained in the axial plane before contrast administration. DWI were obtained by diffusion gradients between 0 and 1000 sec/mm^2^ b-values.

Water molecules show a Brownian motion when placed in a container. This unrestricted motion is called free diffusion [10]. DWI indicate diffusion degree of water molecules in tissues. ADC is the mathematical expression of diffusion as a result of marking the signal loss on the map, which occurs after applying diffusion gradient (the negative logarithm of signal ratios from images obtained using b-values between 0–1000 sec/mm^2^). Relative ADC (rADC) value is defined to minimize effects due to personal factors. Park et al. [11] recommended measurement and comparison with adjacent normal fibroglandular tissue ADC value. rADC value is obtained by dividing lesion ADC value to ADC value of the reference organ [7]. We utilized the fibroglandular tissue of the other breast as the adjacent organ in our study.

### Image Analysis

An ADC map was automatically constructed in a commercially available workstation. Mean ADC values of all lesions were automatically measured by using these maps according to the formula ADC = (lnS0–lnS)/b (signal intensity values are measured as S0 at b = 0 sec/mm^2^ and S at b = 1000 sec/mm^2^). Measurements were performed by placing a region of interest (ROI) of 0.5 mm diameter on lesions. ADC measurements were performed on enhancing or solid parts of the lesions identified in conventional sequences. The ROI did not include normal parenchymal tissue, hemorrhagic or necrotic areas. One radiologist who has experience in breast MRI evaluated the images and performed measurements of ADC and rADC values. A minimum of 3 ADC measurements were performed, and the lowest ADC value was accepted. The ADC value of the contralateral breast at the same level was also measured after ADC measurements were completed for the lesion and ROI of the same diameter was used. The rADC was calculated as the mass ADC value divided by the ADC value of adjacent parenchyma. In addition, the maximum diameters of the tumors on sagittal and axial planes were measured and the mean of these values were expressed as “mean maximum size”.

### Statistical Analysis

The NCSS 10 and SPSS 21 programs were used for statistical analysis. Normality auditing was completed by using the Kolmogorov-Smirnov test, plotting Histogram, Q–Q plot and box plot curves. Continuous variables were presented where applicable as a mean ± standard deviation, minimum-maximum, and percentile. Receiver operating characteristic (ROC) curve analysis was performed for the determination of ADC and rADC cut-off values. Subsequently, the diagnostic values and confidence intervals (CIs) were obtained. The significance level for the study was set as p < 0.05 and bidirectional.

## Results

A total of 88 lesions from 81 patients were evaluated. The mean age of patients was 42.43 ± 11.32 (ranged 16 to 73) years. Fifty-four (66%) of patients were pre-menopausal and 27 (34%) were post-menopausal. A tru-cut biopsy diagnosed seventy-one lesions (80.7%). The remaining 17 lesions (19.3%) were evaluated as BIRADS 3 and remained stable in one-year follow-up which was performed for every six months and accepted as benign breast masses. Pathological results of 34 (38.6%) out of 71 lesions were malignant further broken down into 26 invasive ducal carcinomas, one Paget’s disease, one invasive lobular carcinoma, two inflammatory breast cancers, one papillary carcinoma, two ductal carcinoma in-situ, and one mucinous carcinoma. The patient with Paget’s disease was diagnosed with ductal carcinoma in-situ and the two inflammatory breast cancers were in keeping with invasive ductal carcinoma on histopathologic evaluation. In all of the malignant lesions, one was graded as well-differentiated, 19 were moderately-differentiated, and the remaining 10 lesions were poorly differentiated. Thirty-seven lesions (42%) were histologically benign histopathologically and further broken down as four fatty necroses, six fibrocystic changes, sixteen fibroadenomas, seven granulomatous mastitis, one benign phylloides tumor, one intraductal papilloma, and two hamartomas.

The mean maximum size of benign and malignant lesions were 27 mm (68 mm–6 mm) and 25 mm (50 mm–8 mm), respectively. The mean ADC values were 1.584 × 10^–3^mm^2^/sec (0.833 × 10^–3^mm^2^/sec to 2.460 × 10^–3^mm^2^/sec) for benign lesions and 0.884 × 10^–3^mm^2^/sec (0.830 × 10^–3^mm^2^/sec to 1.490 × 10^–3^mm^2^/sec) for malignant lesions (Table 1) (p < 0.05). The diagnostic performance of ADC value to differentiate malign breast masses from benign masses was as follows: sensitivity 88% (CI 95%; 72–96), specificity 87% (CI 95%; 74–94), positive predictive value (PPV) 81% (CI 95%; 64–91), negative predictive value (NPV) 92% (CI 95%; 80–97), and accuracy is 88% using an ADC cut-off value of 1.04 × 10^–3^mm^2^/sec.

**Table 1:** Distribution of the included breast lesions per histopathologic results and the mean ADC and rADC values of subgroups.

Breast Lesions (n* = 88)	Biopsy results	ADC and rADC value

Malignant lesions (n = 34)	Invasive ductal carcinoma (n = 26) Paget’s disease (n = 1) Invasive lobular carcinoma (n = 1) Inflammatory breast cancer (n = 2) Papillary carcinoma (n = 1) Ductal carcinoma in situ (n = 2) Mucinous carcinoma (n = 1)	Mean ADC value; 0.884 × 10^–3^mm^2^/sec (0.830 × 10^–3^ – 1.490 × 10^–3^)
		Mean rADC value; 0.557 × 10^–3^mm^2^/sec (0.363 × 10^–3^ – 0.866 × 10^–3^)

Benign lesions (n = 54)	Fatty necroses (n = 4) Fibrocystic changes (n = 6) Fibroadenoma (n = 16) Granulomatous mastitis (n = 7) Benign phylloides tumor (n = 1) Intraductal papilloma (n = 1) Hamartoma (n = 2) Benign lesions (n = 17; stable in 1-year follow-up)	Mean ADC value; 1.584 × 10^–3^mm^2^/sec (0.833 × 10^–3^ – 2.460 × 10^–3^)
		Mean rADC value; 0.931 × 10^–3^mm^2^/sec (0.487 × 10^–3^) – 1.511 × 10^–3^)

*n: number of lesions.

**Figure 1:**
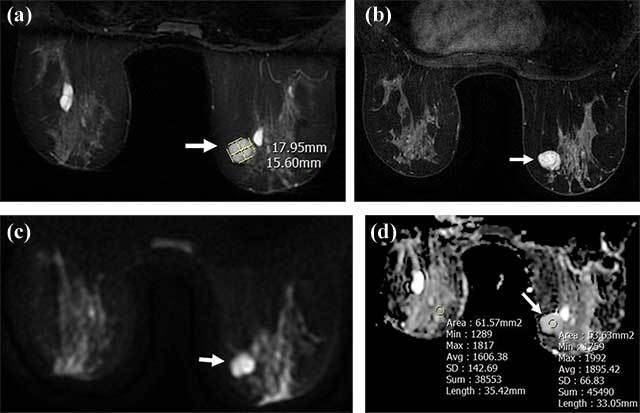
A well-circumscribed 18 × 15.6 mm mass lesion in the right breast upper-inner quadrant hyperintense (arrow) in T2-weighted images **(a)**, homogeneously enhancing (arrow) in T1-weighted post-contrast images **(b)** without diffusion restriction in DWI **(c, d)**; ADC and rADC are 1.895 and 1.179, respectively (d). Tru-cut biopsy results revealed fibroadenoma.

**Figure 2:**
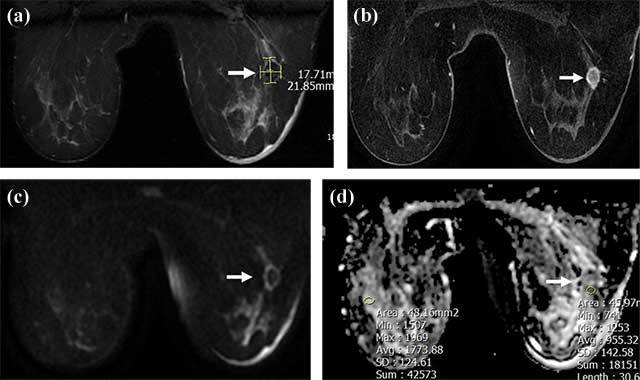
A 18 × 22 mm irregular mass lesion in the right breast lower-outer quadrant, hypointense (arrow) in T2-weighted images **(a)** with rim enhancement (arrow) in contrast images **(b)**, showing diffusion restriction (arrow) in DWI; ADC and rADC values are 0.955 and 0.544, respectively **(c, d)**. Tru-cut biopsy results diagnosed invasive ductal carcinoma.

**Figure 3:**
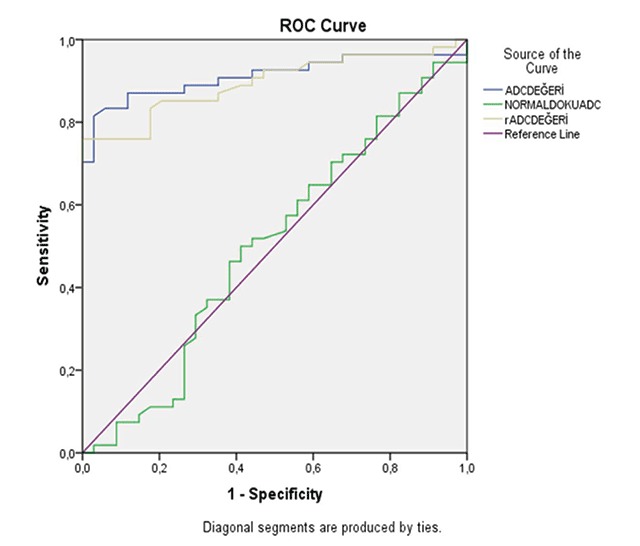
ROC curve analysis. ADC of normal fibroglandular tissue is demonstrated with green, ADC value of mass is demonstrated with yellow and rADC value is demonstrated with blue lines.

The mean ADC value was 1.713 ± 0.32 × 10^–3^mm^2^/sec for normal breast parenchyma (0.915 × 10^–3^mm^2^/sec to 2.453 × 10^–3^mm^2^/sec). The mean rADC values for benign and malignant lesions are 0.931 (0.487–1.511) and 0.557 (0.363–0.866), respectively. Sensitivity was 82% (CI 95%; 65–93), specificity was 83% (CI 95%; 70–92), PPV was 76% (CI 95%; 58–88), NPV was 88% (CI 95%; 75–95), and accuracy was 83% using a 0.639 rADC cut-off. The areas under ROC curves for ADC and rADC were respectively 0.911 ± 0.033 and 0.895 ± 0.034 (Z = 0.69; p = 0.48) (Figures 1, 2 and 3).

## Discussion

Further evaluation with MRI is commonly used for suspicious breast lesions identified in mammographic and sonographic studies. However, the rate of false positivity is high for MRI due to low specificity, and many lesions require biopsy sampling [12,13].

The sensitivity of MRI in breast cancer diagnosis varies between 89%–100% while specificity is reported to be 72% [12,13]. Recently, attempts to increase specificity were undertaken by increasing temporal and spatial resolution and evaluation of kinetic findings altogether. In this respect, the diffusion-weighted sequence was added to routine MRI protocols. There are several studies in literature regarding this issue [14,11,15]. Yabuuchi et al. reported sensitivity and specificity of 92% and 86%, respectively, using DWI.

There are many studies comparing rADC and ADC values for breast masses which were summarized in Table 2. Xie et al. evaluated 66 breast lesions and reported the specificity for ADC and rADC to be 81.5% and 88.9%, respectively; sensitivity and specificity of rADC was significantly higher with respect to ADC [8]. Zao et al. evaluated 48 breast masses, reported 92% specificity for rADC, 84% specificity for ADC (pectoral muscle as the reference organ) and statistically significant difference between rADC and ADC [16]. Ozcan et al. determined ADC and rADC specificity values as 85.71% and 84.13%, respectively, after evaluating 126 breast lesions [9]. Ozcan et al. reported rADC as a useful measurement for differentiation of benign and malignant lesions, but there was no significant difference between rADC and ADC. Sahin et al. reported 100% specificity for ADC and rADC, and 88.5% and 91.4% sensitivity for ADC and rADC, respectively [17]. In a study by El Khouli et al. rADC was defined as normalized ADC [18]. Specificity of rADC (92%) was higher than ADC (72%) in a study conducted with 101 lesions, and rADC was discriminative when some ADC values were equal for benign and malignant lesions [18]. In our study, ADC sensitivity and specificity were 88% and 87%, respectively, while rADC sensitivity and specificity were 82% and 83%, respectively.

**Table 2:** Data of prior studies regarding sensitivity and specificity of ADC and rADC in breast lesions.

Study	n	ADC specificity	rADC specificity

Xie et al. [8]	66	81.5%	88.9%
Zao et al. [16]	48	84%	92%
Ozcan et al. [9]	126	85.71%	84.13%
Sahin et al. [17]	51	100%	100%
El Khouli et al. [18]	101	72%	92%
Yilmaz et al. (Our study)	88	87%	83%

The main factor that decreased the sensitivity and specificity of ADC and rADC were cases of idiopathic granulomatous mastitis (IGM) [19,20], which is consistent with the literature. IGM is a rare chronic inflammatory condition of the breast. It mimics malignancy with restricted diffusion in DWI. We observed restriction in all seven IGM cases in DWI in our study (hyperintense in DAG, hypointense in ADC). Six of these had ADC and rADC prominently lower than threshold value and one at threshold limit. Mean ADC and rADC values were calculated as 0.927 × 10^–3^mm^2^/sec and 0.581, respectively, in IGM cases. High ADC and rADC values were identified in 31 out of 33 BIRADS 3 lesions, diagnosed as fibroadenoma or stable lesions under follow-up; one of the other two lesions had ADC and rADC values below threshold while one had only rADC value below threshold. Evaluation of ductal carcinoma in situ (DCIS) with DWI remains controversial. Many studies indicated a high ratio of false negativity in DCIS diagnosis for low-grade lesions without mass formation [21,22]. The two DCIS cases in our study were both high-grade and no invasive focus was reported after the surgery, and both showed ADC and rADC below the threshold consistent with other malignant lesions.

Consequently, ADC and rADC values, as a means of mathematical expression of DWI, increases sensitivity and specificity of distinction between benign and malignant lesions of the breast.
